# Construction of Chromosome Segment Substitution Lines in Peanut (*Arachis hypogaea* L.) Using a Wild Synthetic and QTL Mapping for Plant Morphology

**DOI:** 10.1371/journal.pone.0048642

**Published:** 2012-11-19

**Authors:** Daniel Fonceka, Hodo-Abalo Tossim, Ronan Rivallan, Hélène Vignes, Elodie Lacut, Fabien de Bellis, Issa Faye, Ousmane Ndoye, Soraya C. M. Leal-Bertioli, José F. M. Valls, David J. Bertioli, Jean-Christophe Glaszmann, Brigitte Courtois, Jean-François Rami

**Affiliations:** 1 CIRAD, UMR AGAP, Montpellier, France; 2 ISRA/Ceraas, Thiès, Sénégal; 3 ISRA, Centre National de Recherche Agronomique, Bambey, Sénégal; 4 Embrapa Recursos Genéticos e Biotecnologia, Brasilia, Distrito Federal, Brazil; 5 Universidade de Brasília, Brasília, Distrito Federal, Brazil; Kansas State University, United States of America

## Abstract

Chromosome segment substitution lines (CSSLs) are powerful QTL mapping populations that have been used to elucidate the molecular basis of interesting traits of wild species. Cultivated peanut is an allotetraploid with limited genetic diversity. Capturing the genetic diversity from peanut wild relatives is an important objective in many peanut breeding programs. In this study, we used a marker-assisted backcrossing strategy to produce a population of 122 CSSLs from the cross between the wild synthetic allotetraploid (*A. ipaënsis*×*A. duranensis*)^4x^ and the cultivated Fleur11 variety. The 122 CSSLs offered a broad coverage of the peanut genome, with target wild chromosome segments averaging 39.2 cM in length. As a demonstration of the utility of these lines, four traits were evaluated in a subset of 80 CSSLs. A total of 28 lines showed significant differences from Fleur11. The line×trait significant associations were assigned to 42 QTLs: 14 for plant growth habit, 15 for height of the main stem, 12 for plant spread and one for flower color. Among the 42 QTLs, 37 were assigned to genomic regions and three QTL positions were considered putative. One important finding arising from this QTL analysis is that peanut growth habit is a complex trait that is governed by several QTLs with different effects. The CSSL population developed in this study has proved efficient for deciphering the molecular basis of trait variations and will be useful to the peanut scientific community for future QTL mapping studies.

## Introduction

Wild relatives of crop plants are sources of useful agriculturally interesting traits that can be tapped to improve the cultivated species [1], [2]. Historically, the transfer of traits from wild to cultivated species was reported to be a labor intensive and time-consuming process that required several backcrosses and was often unsuccessful [3], [4]. The advent of map-based molecular markers and the related quantitative trait locus (QTL) mapping approach allowed the development of new molecular breeding strategies to efficiently exploit the positive alleles remaining in the wild species. The development of interspecific introgression line (IL) libraries monitored by molecular markers allows the representation of the entire wild species genome in a set of lines each of which carries one or few wild donor segments in the genetic background of a cultivated species [3], [5]. These lines are especially useful for mapping complex traits, as they are permanent populations that can be phenotyped in several environments. Moreover, the effects of the alleles of the wild donor parent are evaluated in a homogeneous cultivated genetic background that reduces the interactions between donor alleles [6], [7]. Finally, the introgression lines are useful starting points for breeding [2] and for mapping genes underlying QTLs [6], [8].

Since the pioneering work in tomato [9], several interspecific introgression line libraries with different names but the same genetic construction have been produced for many crops: Chromosome Segment Substitution Lines (CSSLs) for rice [5], [6], Backcross Inbred Lines (BILs) for lettuce [10], Backcross Recombinant Inbred Lines (BCRILs), Introgression Lines (ILs), Near Isogenic Lines (NILs) or QTL-NILs for tomato and barley [9], [11]–[14].

Peanut (*Arachis hypogaea L.*) is an allotetraploid (2n = 4x = 40) tropical legume that is native to South America and has an AB genome. The speciation of cultivated peanut was associated with a change in ploidy level (diploid to allotetraploid), creating reproductive isolation from its wild relatives. This speciation process superimposed with domestication has contributed to the narrow the genetic basis of cultivated peanut.

Peanut wild relatives have long been identified as important sources of interesting traits such as disease resistance [15], [16]. However, the genetic barrier between wild and cultivated peanut species caused by ploidy differences has hampered the use of wild diploid relatives in peanut breeding programs. Nevertheless, disease resistance genes have been successfully transferred from wild diploid peanut species to cultivated tetraploid species [17]–[19].

**Figure 1:**
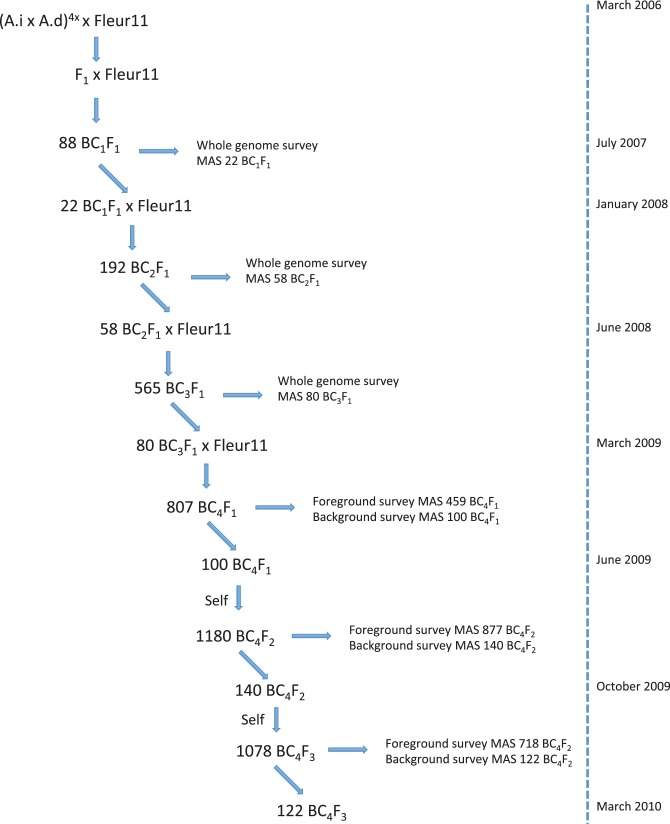
Breeding scheme for the development of the CSSL population.

In the last decade, tremendous efforts have been invested in the development of molecular tools for genetic studies in peanut. Thousands of Simple Sequence Repeat (SSR) markers have been developed and used for genetic map construction and QTL mapping, mainly in intraspecific crosses [20], [21]. In peanut interspecific cultivated×wild crosses, molecular markers have been used as to evaluate the extent of wild chromosome introgressions into the genetic background of cultivated species *a posteriori* [22], or to identify introgressed wild genomic regions that carry disease resistance genes [4], [23]. However, there has been little progress with respect to the integration of molecular markers during the process of alien gene transfer in peanut breeding programs. Thus, there is still a gap to fill by systematizing the use of molecular markers during alien gene transfer and, more generally, by developing permanent interspecific QTL mapping populations such as CSSLs in peanut.

We have recently developed an SSR-based genetic linkage map using a BC_1_F_1_ population derived from the cross between the cultivated Fleur11 variety and the synthetic allotetraploid (*A.* *ipaënsis*×*A. duranensis*)^4x^ [24]. We have also produced an advanced-backcross QTL (AB-QTL) population from the same cross and identified several wild alleles that contribute positive variation to complex traits [25]. Here, as a follow-up to these studies, we report the development of a CSSL population. As a first example of the utility of this population, we have mapped several QTLs involved in peanut morphology. The potential of this CSSL population for deciphering the molecular basis of complex traits is also discussed.

**Table 1:** Selected morphological characteristics of the parents.

Species	Accession	Genome	Ploidy level	Cycle	Growth Habit	Plant Height (cm)	Length of the secondary axis (cm)
*A. ipaënsis*	KG30076	B	2n	Annual	Prostrate	45	50
*A. duranensis*	V14167	A	2n	Annual	Prostrate	20–30	140
*A. hypogaea*	Fleur11	AB	4n	Annual	Erect	16–22	20–25

## Materials and Methods

### Plant Material

The parents of this study were the synthetic wild allotetraploid [*A. ipaënsis* KG30076 (diploid BB genome)×*A. duranensis* V14167 (diploid AA genome)]^4x^ [26] used as the donor parent and the cultivated Fleur11 variety used as the recurrent parent. This synthetic allotetraploid, which combines the A and B genomes of the most probable wild ancestor of cultivated peanut was kindly provided by EMBRAPA in the framework of the Generation Challenge Program (GCP) consortium. Selected agro-morphological characteristics of the wild diploid parents *A. ipaënsis* and *A. duranensis* and the cultivated Fleur11 variety are shown in Table 1. A BC_4_F_3_ population was produced by four successive backcross generations of selected BC_s_ individuals (from BC_1_F_1_ to BC_4_F_1_) to the Fleur11 variety followed by two generations of self-fertilization (from BC_4_F_1_ to BC_4_F_3_). All crosses were performed under greenhouse conditions at the Centre d’Etudes Régional pour l’Amelioration de l’Adaptation à la Sécheresse (CERAAS, Senegal) between 2006 and 2010. The Fleur11 variety was used as female parent to produce the F_1_, BC_1_F_1_ and BC_2_F_1_ generations and as male to produce the BC_3_F_1_ and BC_4_F_1_ generations. The day before each crossing experiment, the plants used as female parent were manually emasculated. The crosses were performed early in the morning from 6 am to 10 am by capping the pollen sacs of the flowers of the male parent on the pistils of the female parent. Flowers that had not been pollinated were removed manually. The crossing scheme and the timeline used to generate the CSSL population are shown in Figure 1.

**Table 2:** Comparison between the observed and expected numbers of heterozygous loci in the population. χ^2^: Chi-square goodness of fit test at 1 ddl.

	All lines			Selected lines		
Generation	Observed	Expected	χ^2^	Observed	Expected	χ^2^
BC_1_F_1_	6630	6496	5.5**	1631	1625	0.04
BC_2_F_1_	5283	5052	14.08***	1438	1649.5	36.15***
BC_3_F_1_	10701	10056.62	47.18***	1353	1508.87	18.40***
BC_4_F_1_	5149	4131.12	267.51***	884	899.75	0.58

### DNA Isolation

At each generation, 3 to 30 seeds from selected BC_n_ (depending on the number of seeds available and on the number of wild chromosome segments to be derived from each individual) were sown in large deep pots in the greenhouse. One seed was sown per pot. Young leaves were harvested from 10–15 day old plants and immediately stored at 4°C on ice before drying. DNA was extracted from 20 mg of dried leaves following a slightly modified mixed alkyltrimethylammonium bromide (MATAB) protocol [27]. Briefly, leaves were ground in quartz sand using a mortar and pestle or using a Mixer Mill (MM301, Retsch) and dissolved in 750 µL of MATAB buffer at 74°C. The samples were incubated for 20 minutes at 74°C and cooled for 5 minutes at room temperature. Then, 750 µL of chloroform-isoamyl alcohol (CIA) (24∶1) was added to each sample. All samples were homogenized by gentle shaking before centrifugation at 12,000 rpm for 20 minutes. The supernatant was collected and the DNA was precipitated with 600 µL of isopropanol. After centrifugation, pellets were washed with 300 µL of 70% ethanol, air dried and dissolved in 500 µL of Tris-EDTA.

**Figure 2:**
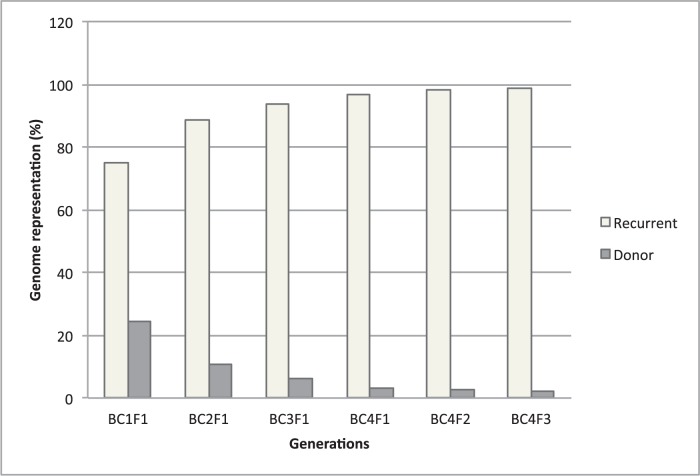
Evolution of donor and recurrent genome representation during the development of the CSSL population.

### Microsatellite Analysis

An SSR-based genetic linkage map based on 88 BC_1_F_1_ individuals was produced previously [24]. A framework map of 115 SSR markers that amplified 147 loci was derived from this map. Compared to the initial map, the framework map offered regular coverage of all of the linkage groups, with an average of one marker per 12 cM. These 115 SSR markers were used to genotype individuals in each backcross generation.

**Figure 3:**
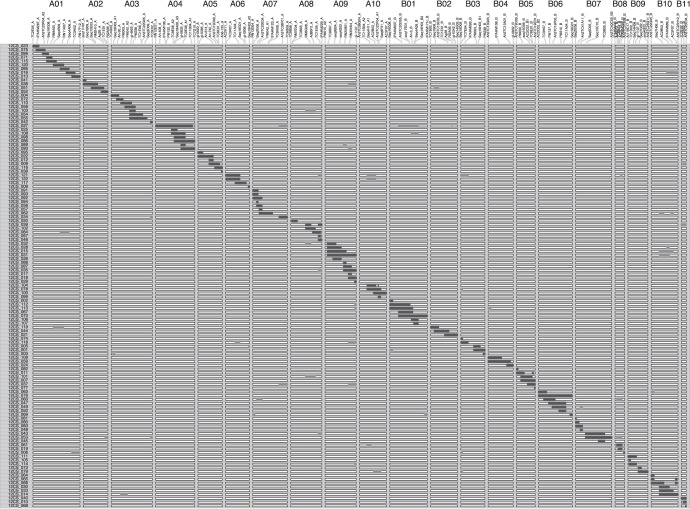
Graphical genotypes of the 122 CSSLs. The 21 linkage groups of the map are named from a01 to a10 (A-genome) and from b01 to b11 (B-genome) and are represented vertically. The marker names are shown on each linkage group. The 122 CSSL are depicted horizontally. The black areas represent the wild target chromosome segments, while the white areas represent the Fleur11 genetic background, and the grey areas represent the wild supernumerary chromosome segments.

**Table 3:** Genotypic features of the progeny of each generation.

Generation	Number of genotyped lines	Number of selected lines	% homozygous Fleur 11 in selected lines	% heterozygous in selected lines	% homozygous AiAd	Mean size of introgressed segments (cM)	Min size of introgressed segments (cM)	Max size of introgressed segments (cM)	Mean number of introgressed segments per line
BC_1_F_1_	88	22	48.68	50.99	0	40.36	2.33	99.65	17.07
BC_2_F_1_	192	58	77.16	21.59	0	35.63	2.35	73.92	11
BC_3_F_1_	565	80	87.31	10.1	1.23	39.23	11.3	115.6	6.43
BC_4_F_1_	807	100	93.66	6.13	0.24	39.15	6.5	115.6	4.06
BC_4_F_2_	1180	140	96.17	2.4	1.4	39.5	5.7	115.6	2.12
BC_4_F_3_	1078	122	97.28	0.14	2.24	39.24	5.7	115.6	1.5

**Table 4:** Variation and descriptive statistics of the traits measured in the CSSL population and the recurrent parent.

	Fleur11						80 CSSLs					
Trait	Nb Plots	Nb plants	Mean	Min	Max	CV(%)	Nb Plots/IL	Nb plants	Mean	Min	Max	CV(%)
GH	60	458	0.00	0.00	0.00	-	6	4169	0.11	0.00	0.87	-
PS	60	458	43.16	34.17	54.40	11.63	6	4169	47.52	31.00	80.40	18.07
PH	60	458	15.23	8.60	21.15	18.39	6	4169	14.83	7.20	24.07	20.90

**Table 5:** Significant line×trait associations observed in the CSSL population.

CSSL Name	Plant nb	Chromosome	GH (%)	PH (%)	PS (%)	YF
12CS_023	55	A01	41.8***	ns	23.5***	0
12CS_016	52	A01	36.9***	−25.7***	18.2**	0
12CS_052	56	A02	ns	17.7*	ns	0
12CS_004	50	A03	20.4***	ns	26.9***	0
12CS_042	48	A03	17.5**	−17.7*	ns	0
12CS_022	54	A05	76.5***	−28***	64.5***	0
12CS_012	51	A05	47.6***	−32.6***	31.9***	0
12CS_009	52	A05	49.9***	−20.9***	41.9***	1
12CS_116	54	A05	39.4***	ns	39.1***	1
12CS_008	57	A06	22.7***	ns	17.2**	0
12CS_091	46	A07	16.4**	−25***	ns	0
12CS_039	52	A08	ns	22.1***	ns	0
12CS_084	48	A08	22.7***	ns	18.6**	0
12CS_104	60	A10	60.1***	ns	53.4***	0
12CS_119	53	B02	ns	19.3**	ns	0
12CS_021	46	B02	ns	19.6**	ns	0
12CS_001	53	B03	ns	21.3***	ns	0
12CS_024	50	B04	36.7***	ns	42.3***	0
12CS_011	55	B05	45.8***	−20.7***	34.8***	0
12CS_007	56	B05	35.2***	ns	25.9***	0
12CS_076	56	B06	29.7***	ns	17.3**	0
12CS_047	57	B06	55.9***	−35.6***	32.6***	0
12CS_050	54	B06	48.4***	−20.4**	40.3***	0
12CS_069	57	B06	ns	31.4***	21.7***	0
12CS_079	53	B09	ns	−18.5**	ns	0
12CS_033	58	B10	60.5***	−16.1*	70.7***	0
12CS_014	53	B10	ns	18.2**	17.3**	0
12CS_010	54	B11	29.0***	ns	33.3***	0

The values for GH (growth habit), PH (plant height) and PS (plant spread) represent the differences between the CSSL line and Fleur11, relative to the value of Fleur11. For the categorical trait YF (flower color), the value 1 corresponds to the yellow flower phenotype. Plant nb: number of plants measured. Chromosome: chromosome carrying the target segment.

**Figure 4:**
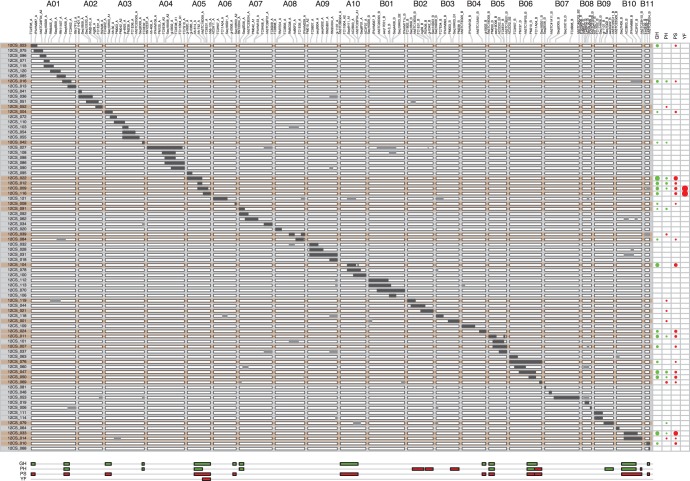
Graphical genotypes of the 80 CSSLs, highlighting significant lines×traits significant association and QTL positions. Graphical genotypes are represented in the same way as in Figure 3. Lines showing significant differences from Fleur11 are highlighted in orange. Relative line effects for each trait are depicted as circles on the right of the graphic. The size of each circle is proportional to its relative effect. Horizontal bars at the bottom of the graphic indicate deduced QTL positions. The colors of the circles and bars represent the direction of the effect. Red = increasing effect, green = decreasing effect.

PCR amplifications were performed in a MJ Research PTC-100 TM thermocycler (Waltham, MA, USA) or in an Eppendorf Mastercycler using 25 ng of DNA in a 10 µl final volume comprising of buffer (10 mM Tris-HCl (pH 8), 100 mM KCl, 0.05% w/v gelatin, and 2.0 mM MgCl_2_), 0.1 µM of the M13-tailed primer, 0.1 µM of the other primer, 160 µM of dNTPs, 1 U of Taq DNA polymerase (Life Technologies, USA), and 0.1 µM of IR700- or IR800-labeled fluorescent M13 primer (MWG, Germany). Touchdown PCR was performed as follows: initial denaturation at 95°C for 1 min; 10 cycles of 94°C for 30 s, Tm (+5°C, −0.5°C/cycle) for 1 min, and 72°C for 1 min; 25 cycles of 94°C for 30 s, Tm for 1 min, and 72°C for 1 min; and a final elongation step at 72°C for 8 min. IR700 or IR800-labeled PCR products were diluted 7-fold and 5-fold, respectively, subjected to electrophoresis in a 6.5% polyacrylamide gel and then sized using the Infra-Red (IR) fluorescence scanning system of the Li-COR sequencer (LI-COR, USA). Images were analyzed using the Jelly 0.1 software (Rami, unpublished) and exported as a data table.

### Marker-assisted Selection of Individuals at Each Backcross Generation

All 115 SSR markers of the framework map were used to genotype a total of 88, 192, and 565 individuals in the BC_1_F_1_, BC_2_F_1_ and BC_3_F_1_ generations respectively. At each generation, the genotyping data were used to select the individuals used to produce further backcross generations. Selection was performed using the CSSL-finder software [28]. To identify a minimum subset of individuals providing an optimal coverage of the cultivated peanut genome with chromosome segments from the wild donor, we selected for individuals with multiple target wild chromosome segments of 30 cM length each, forming contiguous overlapping segments between successive lines, and with the best possible recovery of the genetic background of Fleur11. Using these criteria, 22 individuals were selected and backcrossed in the BC_1_F_1_ generation, 58 in the BC_2_F_1_ generation, and 80 in the BC_3_F_1_ generation.

In the BC_4_F_1_ generation, 807 individuals were genotyped. The genotyping of these individuals was performed in two steps. Because genotypic data were available for the 80 BC_3_F_1_ individuals chosen for developing the BC_4_F_1_ generation, the chromosomal regions that segregated in BC_4_F_1_ individuals were known. Based on this information, foreground selection was first applied to confirm the presence of the target wild chromosome segments in the BC_4_F_1_ individuals; 459 individuals were selected. Background selection was then performed on this subset of individuals at specific regions that still contained undesirable wild segments. A new genotypic dataset that summarized the genotypic information produced in the BC_3_F_1_ generation and those from the foreground and the background selection produced in the BC_4_F_1_ generation was computed. New criteria were added to CSSL-finder to allow redundancy between individuals (i.e., multiple individuals carrying a wild chromosome segment that covers the same genomic region) and to derive only one target wild chromosome segment per line. Finally, using these new criteria, we selected 100 of the 459 BC_4_F_1_ individuals for self-fertilization.

In the BC_4_F_2_ generation, 1180 individuals were genotyped using the foreground selection approach that allowed the identification of 877 individuals carrying a target wild chromosome segment. Background selection was then performed on this subset of individuals. The selection of a final subset of BC_4_F_2_ individuals to be self-fertilized was performed manually. We first selected individuals that carried a unique homozygous or heterozygous target wild chromosome segment in a homogeneous Fleur11 genetic background. This first set of lines did not offer sufficient coverage of the genetic map. In order to cover all regions, we also selected individuals that carried one homozygous or heterozygous target wild chromosome segment and only one other heterozygous wild undesirable chromosome segment elsewhere in the genome. We finally selected among the other plants that had multiple wild chromosome segments, the best individuals with three, four or five segments that covered the genomic regions that had not yet been included. A total of 140 individuals were self-fertilized to produce the BC_4_F_3_ generation.

In the BC_4_F_3_ generation, 1078 individuals were genotyped using the foreground selection approach, which allowed the identification of 718 individuals that carried homozygous target wild chromosome segment. Background selection on this subset of individuals allowed the final selection of the 122 BC_4_F_3_ individuals that were used as our final CSSL population.

### Phenotyping

A subset of 86 CSSLs was selected to provide a minimum set of lines with optimal genome coverage. Six lines could not be included in the phenotyping experiment due to limited seed quantities (12CS_117, 059, 080, 083, 061 and 066) leading to small uncovered regions in linkage groups A06, B04, B07, B08 and B10. The introgressions represented by the final subset of 80 lines covered 88.7% of the genetic map. A field trial involving the 80 selected lines and Fleur11 used as control was conducted from September to December 2011 at the Centre National de Recherche Agronomique (CNRA) in Bambey (14.42°N and 16.28°W), Senegal. The experimental design was an alpha-lattice design with 6 replicates and 6 blocks per replicate. The blocks contained 15 rows of 3 meters each. The 80 CSSL and Fleur11 plants (represented 10 times in each replicate) were arranged in rows of 10 plants. The spacing was 30 cm between plants and 50 cm between rows. Flower color (YF) (orange versus yellow) was rated visually. Peanut growth habit (GH) is usually recorded on a 1–6 scale based on the IBPGR and ICRISAT (1992) descriptors. This recording is well adapted for germplasm description but more difficult to use for QTL mapping because of the qualitative nature of the resulting data. Therefore, in our study, the GH trait was represented by the ratio of the length of the creeping part of a given lateral branch to the total length of the lateral branch. This phenotyping method yielded quantitative values that were more suitable for QTL mapping analyses. Main stem height (PH) was measured from the cotyledonary axil to the terminal bud. Finally, plant spread (PS) was measured as the distance between the widest branch tips. All morphological traits were recorded at 55–60 days after sowing.

### Statistical Analysis

#### Introgression analysis

The frequency of homozygous cultivated Fleur11 and heterozygous wild/cultivated genotypes at each locus was determined for all lines and for the selected lines at each backcross generation (BC_1_F_1_, BC_2_F_1_, BC_3_F_1_ and BC_4_F_1_). The observed homozygous/heterozygous segregation ratio was compared with the expected 0.5, 0.25, 0.125 and 0.0625 ratios in BC_1_F_1_, BC_2_F_1_, BC_3_F_1_ and BC_4_F_1_, respectively, using a chi-square test. The percentage of wild genome at each generation was estimated using the ratio of the total length of the introgressed wild segments to the total length of the genetic map. The total length of the introgressed segments was calculated as the sum of consecutive intervals having heterozygous or homozygous wild genotype plus half the size of each flanking interval with a recurrent parent homozygous genotype. Graphical genotypes were drawn using a script written in R (R Development Core Team, 2010).

#### QTL mapping

Basic statistical analyses were performed for each trait. For QTL detection, an analysis of variance (ANOVA) was first performed with the SAS software to estimate the genotype and replicate effects on each trait following a standard linear model with genotype, replication, block and interaction effects, as follows:
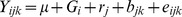
with Y_ijk_ = the observed value for a given trait, µ = mean value of the population, G_i_ = genotype effect, r_j_ = replicate effect, b_jk_ = block within replicate effect and e_ijk_ = residual error. When the analysis revealed a significant difference between genotypes, a Dunnett’s multiple comparison [29] of differences between the CSSLs and Fleur11 was conducted using the GLM procedure in SAS software. We accepted a QTL in a wild chromosome segment when the trait value of a given line was significantly different from that of Fleur11 (P<0.05). QTLs with effects in the same direction on overlapping or flanking segments between contiguous lines were considered to be the same. The relative effect of a wild introgression in a given line was calculated using the least square means (LSMEANS) output of the GLM procedure as follows:



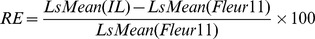



## Results

### Population Development

The F_1_ hybrids between the cultivated Fleur11 variety and the synthetic wild allotetraploid (*A. ipaënsis* KG30076×*A. duranensis* V14167)^4x^ exhibited a good level of fertility. The rate of success of the hybridization in the backcross experiments was also high, ranging from 75% to 80%. However, individuals resulting from inadvertent self-fertilization, identified by molecular analysis, were observed in some cases. Fleur11 is a short cycle variety, and the interval from sowing to the appearance of the first flowers was 21 days on average. The short duration between sowing and flowering required us to limit the number of individuals to be genotyped per backcross family at each generation. On average, 9.6 individuals (min = 3, max = 30) were genotyped per family and per generation. A wide range of phenotypic variation for traits related to growth habit, pod and seed shape and size was observed between the families in the first backcross generations. We were careful to avoid selection based on plant phenotype during all the process of development of the libraries. However, as we advanced in the backcross generations, most of the families exhibited a phenotype similar to that of the recurrent parent, indicating a progressive recovery of the genetic background of the cultivated variety.

#### Heterozygosity and reduction of the wild genome representation in successive backcross generations

When considering all lines, the observed number of heterozygous loci at the backcross generations BC_1_F_1_, BC_2_F_1_, BC_3_F_1_ and BC_4_F_1_ was significantly (P<0.01) higher than expected (Table 2). This could be due to the limited number of lines that were genotyped at each generation. The strategy of population development using marker assisted selection allowed us to significantly (P<0.01) reduce the number of heterozygous loci in BC_2_F_1_ and BC_3_F_1_ generations. This strategy had no significant effect in BC_1_F_1_, due to the low number of lines (22) that were selected, or in BC_4_F_1_, because the heterozygous loci were mainly the target wild chromosome segments. The residual heterozygosity in the CSSLs population was 0.14%.

The average donor genome contribution decreased drastically from BC_1_F_1_ to BC_4_F_1_ (24.6% to 3.0%) and remained stable from BC_4_F_1_ to BC_4_F_3_ (3.0% to 2.3%), during the fixation of target wild chromosome segments (Figure 2).

#### Number and size of the introgressed segments; genome coverage

Marker assisted selection was applied over six generations, from BC_1_F_1_ to BC_4_F_3_, allowing the selection of a set of 122 CSSLs with fixed target wild chromosome segments. The average number of wild donor chromosome segments varied from 17 in BC_1_F_1_ to 1.5 in the CSSL population (Table 3). Among the 122 CSSLs, 72 lines carried one unique donor segment, 37 lines carried two segments, ten lines carried three segments, one line carried four segments and two lines carried five segments. The estimated length of the wild donor segments varied slightly in the different backcross generations and was, on average, 39.2 cM in the CSSL population (Table 3). Among the 122 CSSLs, 42 lines were selected to provide redundancy in some regions of the genome in which several QTLs of agronomic interest had been mapped in a previous study [25]. This was for example the case for LGs a04, a07, a09, b05, b06 and b10 (Figure 3). These lines could be considered potential NILs for these genomic regions.

In the CSSL population, most of the target wild chromosome segments overlap, except those between successive lines in LGs a03, a06, a07 and a08 and in LGs b03, b07 and b09 (Figure 3). The introgressed segments in the whole CSSL population covered 92.1% of the genetic map. Detailed introgression information for each of the 122 CSSL lines is given in supporting information Table S1.

### Phenotypic Analysis

Phenotypic measurements were performed on a subset of 80 CSSLs. Three quantitative traits (GH, PH and PS) and one qualitative trait (YF) were evaluated. The plant growth habit (GH) was measured as the ratio of the length of the creeping part of a given lateral branch to the total length of the lateral branch. The values of the ratio were 0 for an erect growth habit, between 0.16 and 0.5 for decumbent phenotypes, and greater than 0.5 for procumbent phenotypes. The values of the PH and PS traits varied from 7.0 cm to 24.07 cm and from 31.0 cm to 80.4 cm, respectively (Table 4).

For each trait, the mean of the CSSL population was similar to the mean of the recurrent Fleur11 parent (Table 4). The coefficient of variation for the PS trait was higher in the CSSL population than in Fleur11 (18.0% versus 11.0%).

### QTL Detection

For all traits combined, Dunnett’s test revealed a total of 28 lines with significant differences from Fleur11 (Table 5). Among these lines, fifteen had one unique wild chromosome segment, eleven had two wild segments and three had three wild segments. In cases for which a given line had more than one wild segment, it was not obvious how to assign the QTL to a single chromosome segment. We were, however, able to resolve most of these cases by comparison with lines having overlapping segments and similar phenotypes. When the QTL assignment on a single chromosome segment was still ambiguous, QTLs were considered putative in all segments and consequently not assigned. Finally, with these adjustments, 42 QTLs were detected for all traits. In the following paragraphs, we detail the QTL detection per trait.

#### Growth habit (GH)

A total of 20 lines had a significantly different GH compared with Fleur11. The analysis of the line×trait associations allowed the identification of 14 genomic regions containing QTLs. Eleven QTLs were unambiguously mapped to nine linkage groups: a01 (two QTLs), a03 (two QTLs) and LGs a05, a06, a07, b04, b05, b06 and b11 (one QTL each) (Figure 4). For two QTLs, the most likely locations were proposed. The lines carrying these QTLs (12CS_104 and 12CS_033) have a procumbent phenotype and carried each a single wild introgression on LGs a10 and b10 (Figure 4). On LG a10, the wild chromosome segment of line 12CS_104 fully overlaps with that of 12CS_078 and on LG b10, the wild chromosome segment of line 12CS_033 fully overlaps with that of 12CS_014. However, 12CS_078 and 12CS_014 have an erect GH. If we assume that the estimated size of the segments could differ from their real size, because the precise site of recombination is unknown, the most likely locations of the QTLs on LG a10 and b10 are within the regions upstream of the wild segments. The assignment of the remaining QTL was more complicated. For instance, line 12CS_084 carried two wild segments, one of which is located on LG a01 and overlaps with the QTL-carrying segment in line 12CS_016. Thus, these two lines could carry the same QTL. Another wild segment is located on LG a08. This segment partly overlaps with the segments in lines 12CS_101 and 12CS_039. However, these lines have erect GHs. The QTL conferring spreading GH could also be restricted to the non-overlapping region on LG a08. Therefore, this QTL has been considered putative in the a08 linkage group.

In most of the lines, wild alleles at the GH QTLs conferred a decumbent phenotype (ratios between 0.16 and 0.5). However, four lines 12CS_022, 12CS_104, 12CS_047 and 12CS_033, contain wild segments on LG a05, a10, b06 and b10 that conferred a procumbent phenotype (Table 5). Interestingly, 12CS_022 partly overlaps with 12CS_012, 12CS_009 and 12_CS_116, which each have decumbent phenotypes. These results suggest that two QTLs with different effects could be responsible for GH in these regions, one conferring a procumbent phenotype and the other conferring a decumbent phenotype. Alternatively, these lines might share one common QTL but differ at one additional QTL that could be responsible for growth habit differences.

#### Plant height (PH)

A total of 18 lines had main stem height significantly different to that of Fleur11. The analysis of the line×trait associations allowed the identification of 15 genomic regions containing QTLs. Thirteen QTLs were located on ten linkage groups: two QTLs on LG b02, two QTLs on LG b06, 2 QTLs on LG b10 and one QTL each on LGs a01, a03, a05, a07, b03, b05 and b09 (Figure 4). Two QTLs on LG a02 and a08 were considered as putative.

The wild alleles at PH QTLs on LG a01, a03, a05, a07, b05 and b09 were associated with a shorter main stem height, while the two QTLs on LG b02 and one QTL on LG b03 and were associated with an increase in the main stem height (Table 5). Two QTLs with opposing effects were also mapped on LG b06 and b10, respectively.

#### Plant spread (PS)

A total of 20 lines had plant spread values significantly different from that of Fleur11 (Table 5). The line×trait associations allowed the identification of 12 QTLs in 10 linkage groups. Two QTLs were mapped on LG a01, two on LG b06 and one QTL each on LGs a03, a05, a06, a10, b04, b05, b10 and b11 (Figure 4). The plant-spread QTLs co-segregated either with plant growth habit QTLs or with plant height QTLs.

At all QTLs the wild alleles were involved in increasing plant spread. As observed for GH, several lines that have overlapping chromosome segments had also different plant spread. This was the case for successive lines on LG a05 (12CS_022, 12CS_012, 12CS_009 and 12CS_016) and successive lines on LG b10 (12CS_033 and 12CS_014). These results suggest that QTLs with different effects or more than one QTL were responsible for differences in plant spread in these LGs.

#### Flower color (YF)

Two lines, 12CS_009 and 12CS_016, had yellow flowers while Fleur11 had orange flowers. These two lines are successive with contiguous overlapping segments on LG a05 (Figure 4). Thus the genomic region involved in flower color is restricted to the overlapping region between the fragments on LG a05.

## Discussion

### Population Development

The CSSL population we have developed offers broad coverage of the genome of cultivated peanut especially in the context of the large genome size (c. 2800 Mb/1C and 20 linkage groups). The level of coverage of the donor genome of our CSSL population compares favorably to that of sets of introgression lines (IL) in other crop species. In tomato, Eshed and Zamir [9] reported the development of 50 ILs providing a complete genome coverage using the wild species *Lycopersicon pennellii* as donor. In two other studies of tomato IL development, the level of genome coverage was 85% and 97.6%, using *L. hirsutum* [11] and *L. lycopersicoides* [30], respectively, as donors. In barley, 86.6% genome coverage was reported in a set of 59 ILs developed from the cross between the wild species *Hordeum vulgare ssp. spontaneum* and a spring barley cultivar [31]. Lower levels of genome coverage have also been reported. In peanut, a set of 46 ILs covering 30% of the peanut diploid genome has been developed from a cross between the allotetraploid cultivated species *A. hypogaea* and the wild diploid species *A. cardenasii* [22]. In wheat, a set of 97 ILs covering 37.7% of the donor genome has been produced from the cross between a synthetic hexaploid genotype and a Chinese cultivar [32]. In the present study, most of the CSSLs (62.3%) contained a single wild fragment in a homogeneous cultivated genetic background. For the lines that contained more than one fragment, additional backcrossing efforts will be necessary to derive lines that harbor unique wild chromosome segments.

### QTL Mapping

In the present study, as a first demonstration of the utility of the CSSL population, four traits were evaluated in a subset of 80 lines. The line×trait associations allowed the identification of a total of 42 QTLs. Among these QTLs, 37 have been unambiguously assigned to a given genomic region notwithstanding the presence of few additional wild chromosome segments in some lines. Probable locations have been proposed for 2 additional QTLs. This was the case for the GH QTLs on LGs a10 and b10, for which the uncovered regions upstream the wild chromosome segments in lines 12CS_104 and 12CS_033 were proposed as the most probable locations of the QTLs. Among the whole CSSL population (122 lines), one line having wild chromosome segments that cover the QTL region on LG b10 has been identified. This line will be used to confirm the location of the QTL. Finally, three QTLs were putatively assigned to different genomic regions. Additional backcrossing efforts will be necessary for the lines carrying these QTLs to eliminate supernumerary wild chromosome segments and clarify the QTL positions.

#### Growth habit is a complex trait governed by several QTLs with different effects

Peanut growth habit has long been studied, mainly in cultivated×cultivated crosses, and one to four genes have been proposed as responsible for this trait [33]. Genic-cytoplasmic interactions involving at least two cytoplasm types and two genes have also been described [34]. In our recently published study, six QTLs for growth habit have been mapped in an interspecific AB-QTL design involving the same parents as those used in the present study [25]. In the present study, we mapped 14 QTLs, five of which were common with those mapped in the previous study. The difference in the number of QTLs mapped in the two studies may be related to the higher efficiency of QTL detection using the CSSL population. Indeed, in the AB-QTL population, some QTLs, especially those with smaller effects, were likely masked by QTLs with greater effects. Different numbers of mapped QTLs between population types (CSSL versus AB-QTLs) have also been reported in tomato [9] and in rice [35]. In summary, a greater number of QTLs are involved in peanut growth habit in interspecific crosses than in intraspecific crosses. Moreover, in the CSSL population, we observed that lines might have different wild segments carrying QTLs but similar phenotypes. Analyzing these results in an evolutionary context and assuming that the wild species (*A. duranensis* and *A. ipaënsis*) used in this study are the most probable ancestors of cultivated peanut [36], [37], raises new questions about the evolution of cultivated peanut during its domestication. Which QTLs/genes involved in GH have been maintained in the cultivated species? Which ones have been lost? Are similar phenotypes explained by the same or different QTLs? Mapping GH QTLs in intraspecific crosses involving various cultivated varieties and comparing them with QTLs mapped in the CSSL population will shed new light on this issue.

#### QTL effects vary between lines with overlapping wild chromosome segments

In the present study, QTLs with effects in the same direction but of different magnitudes as well as QTLs with opposite effects have been detected in successive lines with overlapping wild chromosome segments. For example, this was the case for the successive lines 12CS_022, 12CS_012, 12CS_009 and 12CS_016, which have different GH effects on LG a05 and for the successive lines 12CS_047, 12CS_050 and 12CS_069, which carry PH QTLs with opposite effects on LG b06 (Table 5). The lines 12CS_047 and 12CS_050 have spreading GH with reduced PH, while 12CS_069 has erect GH with increased PH. Interestingly, on LG b06, the line 12CS_076 carries a wild chromosome segment that overlaps with those of 12CS_047, 12CS_050 and 12CS_069 (Figure 4). This line has spreading GH and PH similar to that of Fleur11. The PH phenotype of 12CS_076 could be caused by QTLs with opposite effects in this LG corresponding to segments of lines 12CS_050 and 12CS_069. Surprisingly, the relative effect of the GH QTL is of lower magnitude in line 12CS_076 than in 12CS_047 or 12CS_050 (Table 5). This result suggests that the QTL involved in increased PH could also have a pleiotropic effect on GH.

Assuming that the QTLs involved in main stem height increase have antagonistic effects on those involved in spreading GH, one could expect that the introgression of these QTLs in cultivated runner-type peanut will modify their growth habit. This could be further confirmed by the introgression of wild segments carrying QTLs involved in increasing PH into the genetic background of a runner-type peanut variety.

### Conclusion

In this study, we produced a population of 122 CSSLs with the aim of broadening the gene pool of cultivated peanut. This population has been efficiently used to dissect the genetic architecture of several morphological traits. Plant growth habit for example, which has long been described as a simple trait governed by one to four genes, was found to be affected by several QTLs with different effects. Our findings confirm the efficiency of the CSSL population type for QTL mapping.

This CSSL population is a public resource, available upon request, and it will be helpful to the peanut scientific community to better understand the molecular bases of the variation of agriculturally interesting traits.

## Supporting Information

Table S1 Introgression information for the 122 CSSL lines. Rows highlighted in grey correspond to the subset of 80 lines characterized in this study.
(XLSX)
